# Effects of Carbon Source on TiC Particles’ Distribution, Tensile, and Abrasive Wear Properties of In Situ TiC/Al-Cu Nanocomposites Prepared in the Al-Ti-C System

**DOI:** 10.3390/nano8080610

**Published:** 2018-08-10

**Authors:** Yu-Yang Gao, Feng Qiu, Tian-Shu Liu, Jian-Ge Chu, Qing-Long Zhao, Qi-Chuan Jiang

**Affiliations:** 1 State Key Laboratory of Automotive Simulation and Control, Jilin University, No. 5988, Renmin Street, Changchun 130025, China; gaoyy16@mails.jlu.edu.cn (Y.-Y.G.); liuts9915@mails.jlu.edu.cn (T.-S.L.); chujg14@mails.jlu.edu.cn (J.-G.C.); zhaoqinglong@jlu.edu.cn (Q.-L.Z.); 2 Key Laboratory of Automobile Materials, Ministry of Education and Department of Materials Science and Engineering, Jilin University, No. 5988, Renmin Street, Changchun 130025, China; 3 Qingdao Automotive Research Institute of Jilin University, No. 1, Loushan Road, Qingdao 266000, China

**Keywords:** in situ nano-TiC, Al matrix nanocomposites, tensile properties, abrasive wear behaviors

## Abstract

The in situ TiC/Al-Cu nanocomposites were fabricated in the Al-Ti-C reaction systems with various carbon sources by the combined method of combustion synthesis, hot pressing, and hot extrusion. The carbon sources used in this paper were the pure C black, hybrid carbon source (50 wt.% C black + 50 wt.% CNTs) and pure CNTs. The average sizes of nano-TiC particles range from 67 nm to 239 nm. The TiC/Al-Cu nanocomposites fabricated by the hybrid carbon source showed more homogenously distributed nano-TiC particles, higher tensile strength and hardness, and better abrasive wear resistance than those of the nanocomposites fabricated by pure C black and pure CNTs. As the nano-TiC particles content increased, the tensile strength, hardness, and the abrasive wear resistance of the nanocomposites increased. The 30 vol.% TiC/Al-Cu nanocomposite fabricated by the hybrid carbon source showed the highest yield strength (531 MPa), tensile strength (656 MPa), hardness (331.2 HV), and the best abrasive wear resistance.

## 1. Introduction

The demands for energy saving and low fuel consumption in the structural fields and heat sink applications have inspired the developments of the lightweight Al alloys and corresponding Al matrix composites [1,2,3,4,5,6]. The Al matrix composites with ceramic particles reinforcements show more excellent thermal stability, mechanical performance and wear resistance than the Al alloys [7,8,9,10,11,12]. The commonly used ceramic particle enhancers for Al matrix composites are TiC, TiB_2_, SiC, and B_4_C ceramic particles [13,14,15,16,17,18,19]. The TiC particles showed low mismatch with Al matrix and obtained excellent Al-TiC interface bonding without the generation of interfacial reaction products [20,21,22,23,24,25]. The superior Al-TiC interface provided good adhesion between the TiC particles and Al matrix, which contributed to decrease the thermal boundary resistance, resist the plastic deformation and increased the strength and hardness of the Al matrix [4,26,27]. It is necessary to mention that, the Al-Cu composites reinforced with nano-sized ceramic particles showed finer α-Al grains, higher tensile strength and ductility, higher creep resistance, and better sliding wear resistance than those of the composites reinforced with the micron-sized particles [7,28,29]. As reported, the in situ TiC/Al-Cu nanocomposites obtained simultaneous increases in tensile strength and ductility at high temperatures (ranging from 298 K to 573 K) compared to the Al-Cu matrix alloys [23,25]. Tian et al. found that, the 0.5 wt.% TiC/Al-Cu nanocomposite showed much better sliding wear resistance than the Al-Cu alloy, with the highest increase of 83.5% in relative wear resistance at 453 K and applied load of 20 N [7]. The nano-TiC particles are very effective and promising enhancers for the Al-Cu alloys.

The most challenge work for the Al matrix nanocomposite is how to obtain homogenous distribution of the nanoparticles, which greatly affect the thermal, electrical, and mechanical properties of the nanocomposites [8,19,27]. Currently, the TiC/Al nanocomposites are prepared by the ex and in situ processing routes, such as mechanical alloying, stir casting, powder metallurgy, spark plasma sintering, combustion synthesis, etc. [30,31,32,33,34,35,36]. However, these ex situ methods possess some drawbacks, such as poor interface bonding, uneven distributions, and interface contamination [34,37]. Zhou et al. modified TiC nanoparticles by deposition Ni coating on the TiC surface to improve the wetting between TiC and Al matrix, and the 0.8 wt.% TiC/Al–Cu nanocomposite prepared by stir casting obtained the highest tensile strength (573 MPa) [34]. The distribution of the TiC nanoparticles in the Al matrix composites prepared by methods of mechanical alloying, powder metallurgy and spark plasma sintering were largely depended on the ball milling parameters, such as milling times, speed and the weight ratio of ball to powders [4,5,31,35]. Nemati et al. prepared the Al-4.5 wt.% Cu-TiC nanocomposites by fabrication route of high energy attritor ball milling and hot sintering to obtain uniformly-dispersed TiC nanoparticles [31]. When the TiC nanoparticles content is higher than the critical level (5 wt.%), the TiC nanoparticles agglomerations increased, which lowered the strengthening effects [31]. The in situ TiC/Al nanocomposites, which were synthesized in Al-Ti-CNTs systems by the method of combustion synthesis and hot pressing, have obtained a relatively ideal distribution of nano-TiC particles and excellent Al-TiC interface bonding [25,38]. The combined method of combustion synthesis, hot pressing, and hot extrusion can further improve the nanoparticles distribution in the synthesized nanocomposites and densify the nanocomposites. The in situ TiC/Al-Cu-Mg nanocomposites synthesized by this combined method showed superior room temperature tensile properties with superior yield strength (404 MPa), tensile strength (601 MPa), and fracture strain (10.8%) [25]. However, the CNTs shows strong tendency to form agglomerations due to the strong van der Waals force. In the ball-milling process, the CNTs can be easily tangled and twisted together. As reported, the CNTs’ clusters in the raw reaction powders led to the uneven distribution of precipitated nano-TiC particles in the synthesized nanocomposites [25]. Chen et al. took the combined methods of the high energy ball milling and the solution ball milling to obtain the homogenously distributed CNTs on the Al powders [39]. Najimi et al. took a mixed ball-milling mode (200 rpm, 2 h + 1000 rpm, 1 h) to improve the CNTs distribution in the mixed powders, which successfully improved the mechanical properties of prepared composites [40]. It is well known to us that the high-speed ball-milling device is usually very expensive and the cost of CNTs is also high. Carbon black (C black) is another commonly used and easily dispersed carbon source with fine size and low cost. It is reported that the TiC particles synthesized by carbon source of C black show larger average sizes compared to that synthesized by CNTs [41,42]. The increase in TiC particles sizes might weaken the van der Walls force between the nanoparticles and impair the tendency of the nano-TiC particles to form clusters in the synthesized nanocomposites. Therefore, we chose the hybrid carbon source (C black + CNTs) to improve the nano-TiC particle distribution in the synthesized TiC/Al-Cu nanocomposites.

In this study, in situ TiC/Al-Cu nanocomposites were synthesized in Al-Ti-C systems with various carbon sources (C black, hybrid C/CNTs and CNTs) by the combined method of combustion synthesis, hot pressing and hot extrusion. The effects of carbon source on the nano-TiC particles distribution, tensile, and abrasive wear properties of the TiC/Al-Cu nanocomposites were studied. This work aims at providing a simple and low-cost way to obtain the in situ TiC/Al-Cu nanocomposites with high strength and abrasive wear resistance. 

## 2. Experimental Procedure

**Figure 1:**
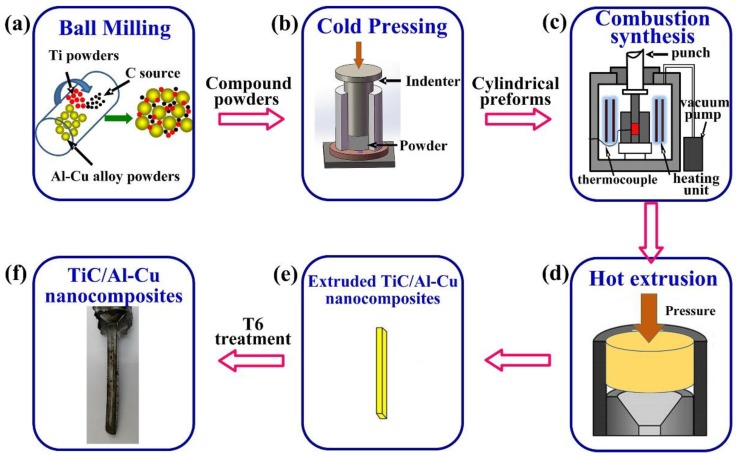
The process schematic diagrams for preparation the in situ TiC/Al-Cu nanocomposites, (**a**) ball milling process of the raw powders, (**b**) cold pressing into the cylindrical compacts, (**c**) fabrication the TiC/Al-Cu nanocomposites by combustion synthesis and hot pressing, (**d**) hot extrusion, (**e**) T6 heat treatment, and (**f**) TiC/Al-Cu nanocomposites.

The raw powders were Al-Cu (Al-4.7 wt.% Cu-0.32 wt.% Mg-0.65 wt.% Mn-0.44 wt.% Si) alloy powders (~13 μm, 99% purity), Ti powders (~25 μm, 99.5% purity), C black (~40 nm, 99% purity), and CNTs (~20–100 μm in length and 10–20 nm in diameter, 95.0% purity). The nominal compositions of TiC/Al-Cu nanocomposites were presented in Table 1. Figure 1 shows the process schematic diagrams for preparation the in situ TiC/Al-Cu nanocomposites. The mixed Al alloy powders, Ti powders, and carbon powders (C black/CNTs) were ball milling at a speed of 50 rpm for 18 h. The molar ratio of Ti/C was 1:1. The ball-milled powders were cold pressed into the cylindrical compacts (ϕ 45 mm × 36 mm). The fabrication of the TiC/Al-Cu nanocomposites were performed in a vacuum furnace at a heating speed of about 30 K/min. When a sudden jump in temperature observed, the combustion synthesis reaction proceeded and the compact was densified by applying an axial press (~50 MPa for 20 s). The Al-Cu matrix alloy was prepared by the method of hot press sintering at 873 K for 60 min. The hot extrusion was performed on the sintered Al-Cu alloy and TiC/Al-Cu nanocomposites with an extrusion ratio of 19:1 at 833 K. The extruded sheets were subjected to T6 heat treatment, which included solution treatment (778 K for 2 h), quenching in water, and artificial aging (433 K for 18 h).

The microstructure of the synthesized TiC/Al-Cu nanocomposites was observed by scanning electron microscopy ((SEM, Evo18, Carl Zeiss, Oberkochen, Germany) and TEM (JEM 2100F, JEOL, Tokyo, Japan). The morphology of the nano-TiC particles was observed by FESEM (JSM-6700F, JEOL, Tokyo, Japan). The bulk nanocomposites were dissolved in 18 vol.% HCl aqueous solution to remove the Al matrix. About one thousand nano-TiC particles were randomly selected to count the average sizes. The preparation of TEM foils were firstly ground to 60 μm by mechanical polishing and then thinned by twin-jet electrical polishing in a mixed solution (10% perchloric acid + 90% ethanol) at 15 V and 243 K.

The specimens for the tensile and the abrasive wear tests were machined from the nanocomposites along the extrusion direction. The dog-bone shaped tensile samples had a gauge length, gauge width, and gauge thickness of 10 mm, 4 mm, and 2 mm, respectively. The room tensile tests were performed using a servo hydraulic materials testing system (MTS 810, MTS Systems Corporation, Minneapolis, MN, USA) at a strain rate of 3 × 10^−4^ s^−1^. The abrasive wear tests were conducted on a ML-30 wheeled abrasion tester with the sliding distance of 120 m and the applied load of 5 N, 15 N, and 25 N. The abrasive test was carried on the surface of extruded samples parallel to the extrusion direction with dimensions of 40 mm × 5 mm. The abrasive Al_2_O_3_ papers with the abrasive particle sizes of 6.5 μm, 13 μm, and 23 μm were selected as the counter face. The actual density of the extruded samples was tested by the Archimedes method. The synthesized Al-Cu matrix alloy and nanocomposites obtained a high relative density (>100%), which meant that the nanocomposites were fully densified by the hot extrusion process. The volume loss during abrasive wear testing was determined by using mass loss divided by actual density. The abrasive wear resistance was defined by the ratio of the wear rate of the Al-Cu matrix alloy to that of the TiC/Al-Cu nanocomposites. The abrasive wear resistance of the Al-Cu alloy was set to the 1.00. The tensile and abrasive wear tests were repeated three times to characterize the tensile and abrasive wear properties of the samples. The micro-hardness of the specimens was characterized by a Vickers hardness tester (1600-5122VD, Buehler, Feasterville, PA, USA), using a dwell time of 10 s and an applied load of 5 N for 10 times.

## 3. Results and Discussion

### 3.1. Microstructure and Mchanical Properties

**Figure 2:**
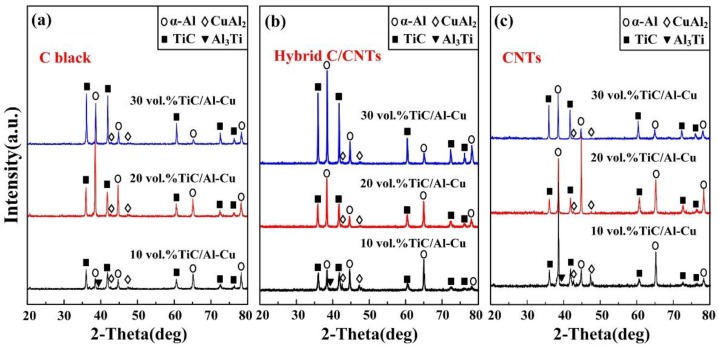
The XRD patterns of the synthesized TiC/Al-Cu nanocomposites fabricated by carbon sources of (**a**) pure C black, (**b**) hybrid C/CNTs, and (**c**) pure CNTs.

Figure 2 shows the XRD detection results of TiC/Al-Cu nanocomposites prepared in the Al-Ti-C system by various carbon sources. It can be observed that the phase compositions of prepared TiC/Al-Cu nanocomposites are mainly composed of Al, TiC, CuAl_2_, and Al_3_Ti. When a lower content of reactants of Ti and C in the Al-Ti-C system occurs, the reaction could not proceed sufficiently and intermediate products remains. As shown in Figure 2a–c, the residual Al_3_Ti phase could be observed in the 10 vol.% TiC/Al-Cu nanocomposites prepared by different carbon sources. The actual volume fractions of nano-TiC particles in the nanocomposites 10B (8.9 vol.%), 10H (9.0 vol.%), and 10C (9.2 vol.%) were lower than the designed content (10 vol.%). With the reactants increasing, pure reaction products were obtained in the fabricated 20 vol.% and 30 vol.% TiC/Al-Cu nanocomposites, and the actual volume fractions of the nano-TiC particles were very close to the target values.

**Figure 3:**
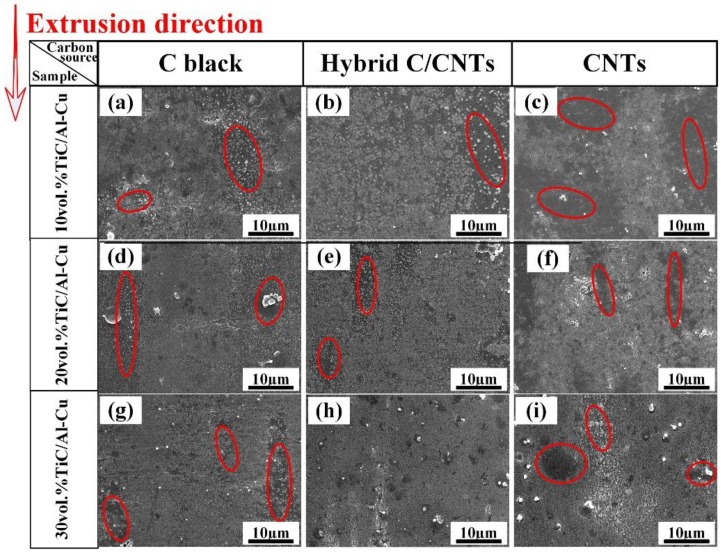
SEM micrographs of the extruded TiC/Al-Cu nanocomposites fabricated by carbon sources of pure C black, hybrid C/CNTs, and pure CNTs. (**a**) nanocomposite 10B, (**b**) nanocomposite 10H, (**c**) nanocomposite 10C, (**d**) nanocomposite 20B, (**e**) nanocomposite 20H, (**f**) nanocomposite 20C, (**g**) nanocomposite 30B, (**h**) nanocomposite 30H and (**i**) nanocomposite 30C.

Figure 3 shows the SEM micrographs of the extruded TiC/Al-Cu nanocomposites fabricated by carbon sources of C black, hybrid C/CNTs, and CNTs. As the nano-TiC particles content increased, non-homogenous distribution regions of nano-TiC particles (labeled by the red circles) were obviously reduced, which means that the distribution of the nano-TiC particles was improved in the nanocomposites. The nanocomposites 30B, 30H, and 30C obtained relatively uniform distribution of nano-TiC particles, as shown in Figure 3g–i. On the other hand, the nanocomposites fabricated by the hybrid C/CNTs (nanocomposites 20H and 30H) showed more homogenous distributions than those of the nanocomposites fabricated by the pure C black (nanocomposites 20B and 30B) and the pure CNTs (nanocomposites 20C and 30C).

**Figure 4:**
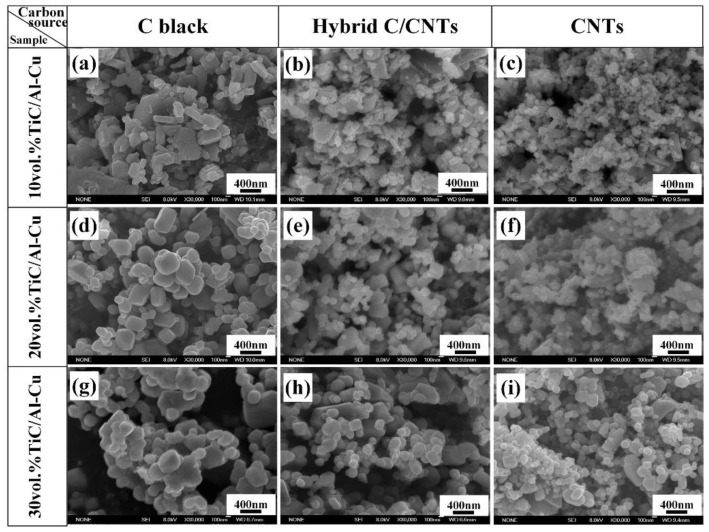
FESEM images of extracted nano-TiC particles from TiC/Al-Cu nanocomposites fabricated by carbon sources of pure C black, hybrid C/CNTs, and pure CNTs. (**a**) nanocomposite 10B, (**b**) nanocomposite 10H, (**c**) nanocomposite 10C, (**d**) nanocomposite 20B, (**e**) nanocomposite 20H, (**f**) nanocomposite 20C, (**g**) nanocomposite 30B, (**h**) nanocomposite 30H and (**i**) nanocomposite 30C.

**Figure 5:**
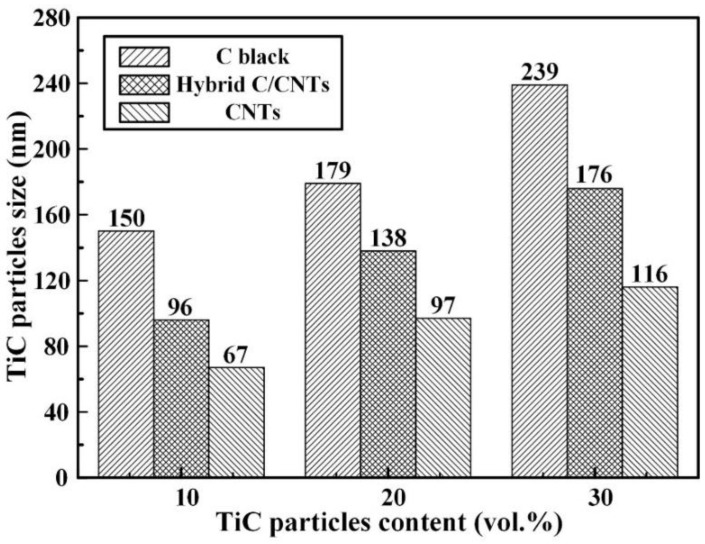
The average sizes of nano-TiC particles in TiC/Al-Cu nanocomposites fabricated by carbon sources of pure C black, hybrid C/CNTs, and pure CNTs.

Figure 4 and Figure 5 show the FESEM images and average sizes of nano-TiC particles fabricated in Al-Ti-C systems with carbon sources of pure C black, hybrid C/CNTs, and pure CNTs. The nanocomposite 10C obtained the smallest sized nano-TiC particles (67 nm). With the increase in nano-TiC particles content, the average sizes of nano-TiC particles increased from 67 nm in nanocomposite 10C, 97 nm in nanocomposite 20C to 116 nm in nanocomposite 30C. When the carbon sources in Al-Ti-C systems transferred from C black, hybrid C/CNTs to CNTs, the average sizes of generated nano-TiC particles decreased obviously. By using the hybrid carbon black/CNTs, the nanocomposite 30H showed moderate nano-TiC particles size of 176 nm between the nanocomposite 30B (239 nm) and the nanocomposite 30C (116 nm).

In the Al-Ti-C reaction system, Al powders in the original reactants firstly melted and then Al_3_Ti phase formed by Al-Ti reaction. As the temperature increased, Al_3_Ti phase dissolved and Al-Ti binary liquid formed. With the heating temperature further increasing, Al-Ti-C ternary liquid phase formed with the decomposition and dissolve of carbon source. With the concentration of [C] reached the critical point, the [Ti]-[C] reaction ignited and nano-TiC particles precipitated. The carbon decomposition accelerated and the combustion reaction proceeded sufficiently by the great amount of heat generated by the [Ti]-[C] reaction. Considering the higher chemistry activity and higher specific area of CNTs, the Al-Ti-C liquid phase could occur at a lower temperature with the carbon source of CNTs than those of C black and hybrid C/CNTs. Therefore, the Al-Ti-C system with the carbon source of CNTs reacted at a lower temperature. Furthermore, the rapid decomposition of the CNTs guaranteed the shorter combustion reaction time of Al-Ti-C system when compared to the carbon sources of C black and hybrid C/CNTs. According to the index functional relation between the ceramic particles growth and the combustion temperature, the lower combustion temperature assured the smaller sizes of precipitated nano-TiC particles [26,41]. As a consequence, the sizes of generated nano-TiC particles decreased gradually with the carbon sources transferring from CNTs, hybrid C/CNTs to C black.

However, the twisting and tangling of CNTs would cause the non-homogenous distribution of the rich regions of dissolved [C] and, subsequently, nano-TiC particles’ precipitation in the Al matrix. Moreover, the van der Waals forces between in the nano-TiC particles became more favorable with the nano-TiC particles sizes decreasing when carbon sources transferring from C black, hybrid C/CNTs, to CNTs. As a consequence, the nano-TiC/Al-Cu nanocomposites synthesized by a carbon source of CNTs exhibited terrible dispersions of nano-TiC particles, as shown in Figure 3c–i. In contrast to the CNTs, the C black with lower chemistry activity and decomposition rate would lead to the uneven distribution of [C] regions. These uneven [Ti]-[C] reactions occurred which resulted in the abnormal growth and non-ideal dispersion of precipitated nano-TiC particles. Additionally, the great amount of heat released by uneven reaction regions might cause the formation of voids, which is reflected by the lower actual density of TiC/Al-Cu nanocomposites synthesized by a carbon source of C black, as observed in Table 2. In this paper, we took the hybrid C/CNTs to weaken the twisting and tangling of CNTs and obtain relative uniform distribution of carbon source in the Al-Ti-C system. Finally, the TiC/Al-Cu nanocomposites fabricated by the carbon source of hybrid C/CNTs exhibited a more homogenous distribution of nano-TiC particles.

**Figure 6:**
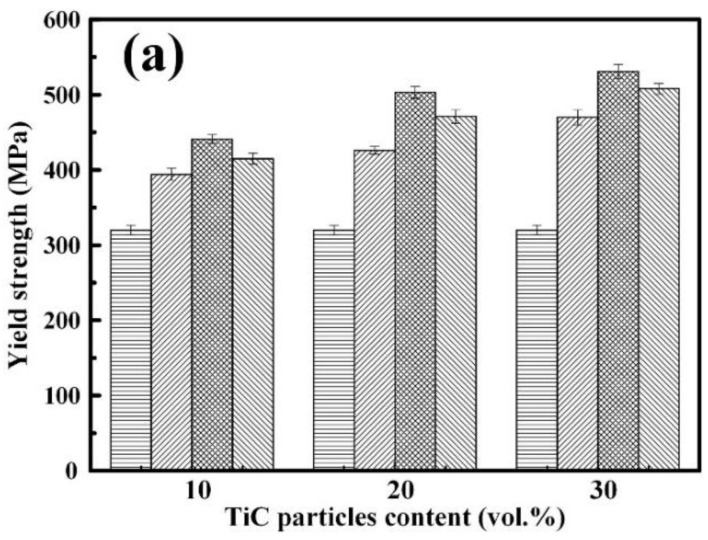

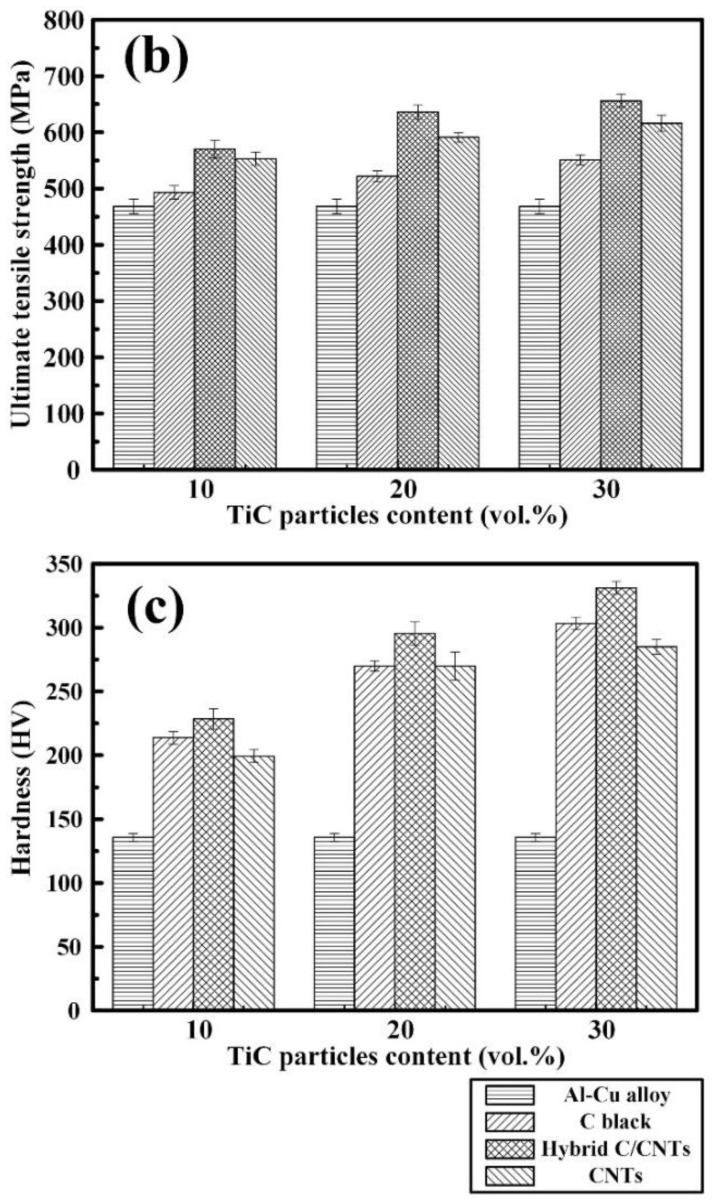
The (**a**) yield strength, (**b**) tensile strength, and (**c**) hardness of TiC/Al-Cu nanocomposites fabricated by carbon sources of pure C black, hybrid C/CNTs, and pure CNTs.

Figure 6 shows the yield strength (*σ*_0.2_), tensile strength (*σ*_UTS_), and hardness (HV) of the Al-Cu alloy and the TiC/Al-Cu nanocomposites. The room tensile properties (*σ*_0.2_, *σ*_UTS_, and fracture strain (*ε*_f_)) and hardness are presented in Table 2. As observed in Figure 6, the TiC/Al-Cu nanocomposites showed much better *σ*_0.2_, *σ*_UTS_ and HV compared to the Al-Cu alloy. As the nano-TiC particles content increased, the *σ*_0.2_, *σ*_UTS_, and HV of the nanocomposites increased significantly. The nanocomposites 30B, 30H, and 30C showed the superior *σ*_UTS_ and hardness of 551 MPa and 303.3 HV, 656 MPa, and 331.2 HV, and 616 MPa and 285.1 HV, respectively. On the other hand, the nanocomposites fabricated by carbon source of the hybrid C/CNTs (nanocomposites 20H and 30H) showed better *σ*_0.2_, *σ*_UTS_, HV, and actual density than those of the nanocomposites fabricated by C black (nanocomposites 20B and 30B) and CNTs (nanocomposites 20C and 30C). It can be inferred that the number of nano-TiC particles in nanocomposite 30B is less than that in the nanocomposites 30H and 30C due to the larger nano-TiC particle sizes. Compared to the nanocomposite 30C, the nanocomposite 30H obtained a more homogenous distribution of nano-TiC particles. The nanocomposite 30H might obtain better Orowan strengthening effects than those of nanocomposites 30B and 30C attributed to the higher number and more homogenous distribution of nano-TiC particles. The nanocomposite 30H obtained the best comprehensive tensile properties and hardness with *σ*_0.2_, *σ*_UTS_, *ε*_f_, and HV of 531 MPa, 656 MPa, 3.0%, and 331.2 HV, respectively.

**Figure 7:**
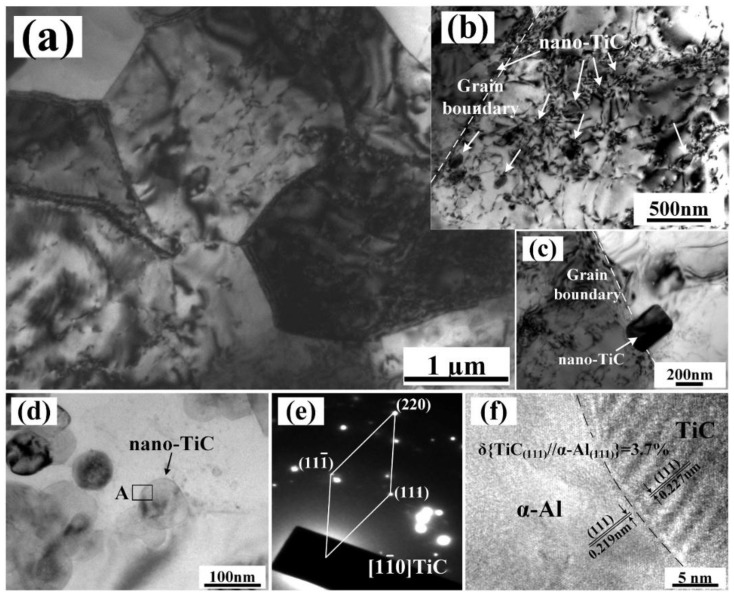
TEM micrographs of 20 vol.% TiC/Al-Cu nanocomposites fabricated by the hybrid carbon source. (**a**–**c**) microstructure images, (**d**) nano-TiC particles, and (**e**,**f**) corresponded selected-area electron diffraction (SAED) patterns and HRTEM image of area A.

Figure 7 shows the TEM micrographs of the nanocomposite 20H after hot extrusion and T6 heat treatments. Figure 7a shows the fine sized α-Al grains of the nanocomposite 20H. Figure 7b,c show the nano-TiC particles distributed in the interior and at the grain boundaries of α-Al grains. Figure 7d,e show the morphology and corresponded selected-area electron diffraction (SAED) of the generated nano-TiC particles. As shown in Figure 7f, the in situ nano-TiC particles showed a low mismatch with Al matrix (3.7%). Attributed to the good wetting ability of in situ nano-TiC particles with molten Al, some nano-TiC particles were captured by the growing α-Al grains, which were observed in the interior of α-Al grains, as shown in Figure 7b. Additionally, the nano-TiC particles, which were not captured by the rapidly advancing liquid-solid interface, were eventually distributed at the grain boundaries, as shown in Figure 7c. The nano-TiC particles, distributed at grain boundaries and within the α-Al grains, impeded the dislocation motions. The dislocation networks and dislocation walls formed, as seen in Figure 7a,b. As a result, the nano-TiC particles increased the plastic deformation resistance of the nanocomposites, which is reflected by the improved strength and hardness of the nanocomposites. Attributed to the most homogeneous distribution of nano-TiC particles and high nano-TiC particles content (30 vol.%), the nanocomposite 30H exhibited the best Orowan strengthening effects and highest tensile strength and hardness.

### 3.2. Abrasive Wear Behaviors

**Figure 8:**
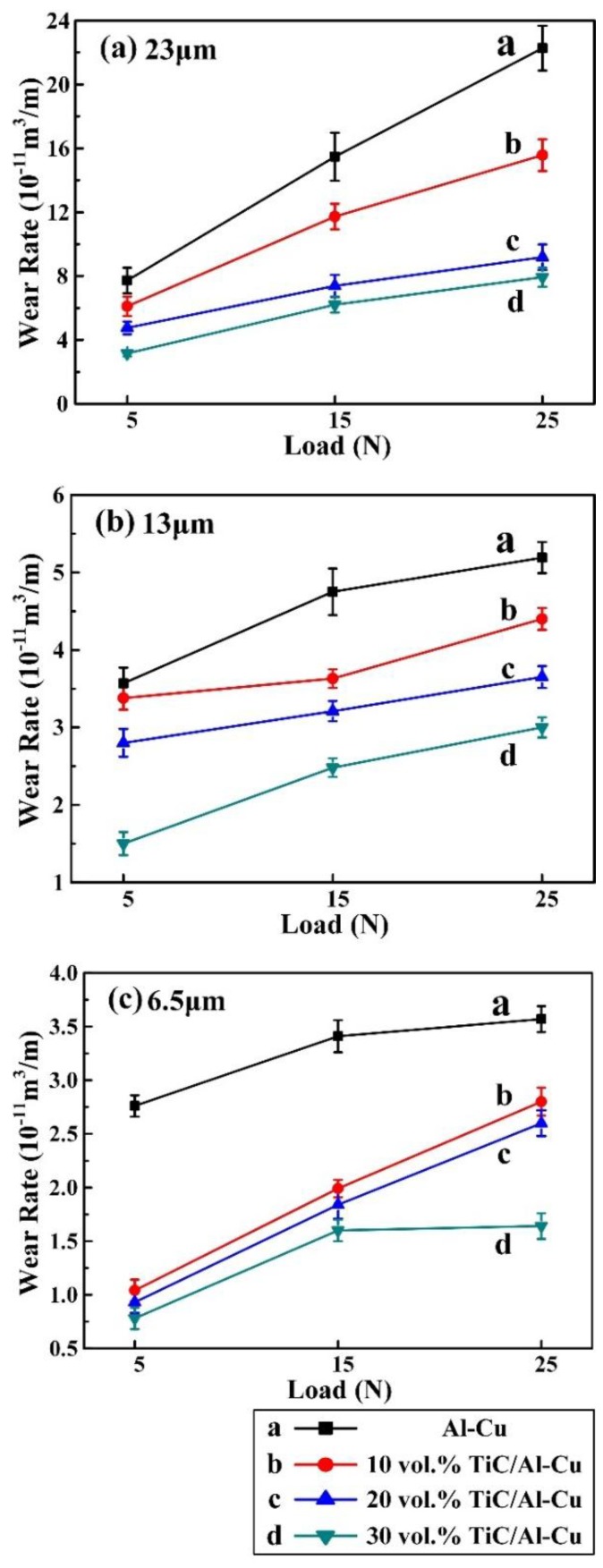
Variations of wear rate with applied load for the Al-Cu alloy and TiC/Al-Cu nanocomposites (fabricated by the hybrid C/CNTs) under the abrasive Al_2_O_3_ particle sizes of (**a**) 23 μm, (**b**) 13 μm, and (**c**) 6.5 μm.

Figure 8 shows the variations of wear rate with applied load (5 N, 15 N, and 25 N) for the Al-Cu matrix alloy and in situ TiC/Al-Cu nanocomposites (nanocomposites 10H, 20H, and 30H) against various Al_2_O_3_ particle sizes. As the applied load increased, the wear rates (WR) of the Al-Cu matrix alloy and the nanocomposites increased. As shown in Figure 8a, with the applied load increasing from 5 N, to 15 N, to 25 N, the WR (10^−11^ m^3^/m) of the Al-Cu matrix alloy increased from 7.72, to 15.48, to 22.27 against the abrasive particle size of 23 μm. The TiC/Al-Cu nanocomposites showed much lower WR than those of the Al-Cu matrix alloy. The WR of the nanocomposites decreased obviously with the increase in the nano-TiC particles content. As the nano-TiC particles content increased from 10 vol.%, 20 vol.%, to 30 vol.%, the hardness of the nanocomposites increased from 228.5 HV in the nanocomposite 10H, 295.6 HV in the nanocomposite 20H to 331.2 HV in the nanocomposite 30H, while the WR (10^−11^ m^3^/m) decreased from 15.57 in the nanocomposite 10H, 9.19 in the nanocomposite 20H to 7.93 in the nanocomposite 30H with the applied load of 25 N and the abrasive Al_2_O_3_ particle size of 23 μm. The abrasive wear resistance of the nanocomposite 30H was 1.81 times higher than that of Al-Cu alloy under the applied load of 25 N and abrasive Al_2_O_3_ particle size of 23 μm. On the other hand, the WR of the Al-Cu matrix alloy and the nanocomposites decreased when the abrasive Al_2_O_3_ particle sizes decreased. The WR of the nanocomposite 30H decreased from 7.93, to 3.01, to 1.64 with the decreases in the abrasive Al_2_O_3_ particle sizes from 23 μm, to 13 μm, to 6.5 μm under the applied load of 25 N. With the applied load of 5 N and abrasive Al_2_O_3_ particle size of 6.5 μm, the WR of nanocomposite 30H was 0.78 (10^−11^ m^3^/m), and the abrasive wear resistance was 2.54 times higher than that of the Al-Cu alloy.

**Figure 9:**
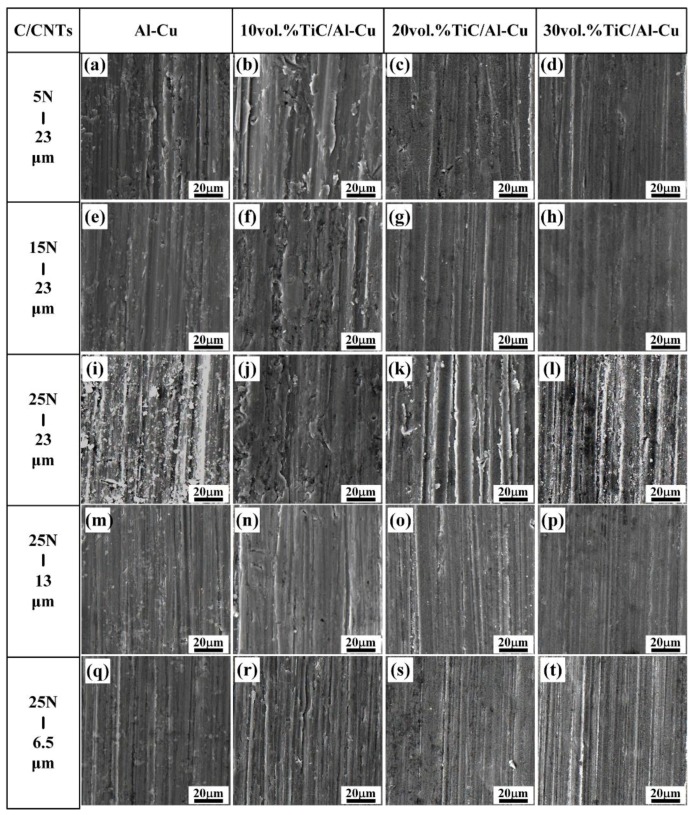
The SEM micrographs of worn surface of the Al-Cu alloy and TiC/Al-Cu nanocomposites (fabricated by the hybrid C/CNTs) under various abrasive Al_2_O_3_ particle sizes and applied loads. (**a**) Al-Cu alloy, (**b**) 10H, (**c**) 20H and (**d**) 30H under tested load of 5 N and abrasive Al_2_O_3_ particle size of 23 μm; (**e**) Al-Cu alloy, (**f**) 10H, (**g**) 20H and (**h**) 30H under tested load of 15 N and abrasive Al_2_O_3_ particle size of 23 μm; (**i**) Al-Cu alloy, (**j**) 10H, (**k**) 20H and (**l**) 30H under tested load of 25 N and abrasive Al_2_O_3_ particle size of 23 μm; (**m**) Al-Cu alloy, (**n**) 10H, (**o**) 20H and (**p**) 30H under tested load of 25 N and abrasive Al_2_O_3_ particle size of 13 μm; (**q**) Al-Cu alloy, (**r**) 10H, (**s**) 20H and (**t**) 30H under tested load of 25 N and abrasive Al_2_O_3_ particle size of 6.5 μm.

Figure 9 shows the worn surface of the Al-Cu alloy and the nanocomposites 10H, 20H, and 30H. As observed, the nanocomposites showed much smoother worn surface compared to the Al-Cu alloy. As mentioned above, the nanocomposites with higher nano-TiC particles content obtained higher HV, which decreased the contacting area between the abrasive Al_2_O_3_ particles and the samples. On the other hand, the nano-TiC particles helped to constrain the plastic deformation of Al matrix, impeded the penetrating of abrasive particles and reduced the cutting efficiency of abrasive particles, which helped to increase the abrasive wear resistance of the nanocomposites [26]. As the nano-TiC particles content increased from 10 vol.%, 20 vol.%, to 30 vol.% in the nanocomposites, the micro-ploughing efficiency of the abrasive Al_2_O_3_ particles decreased and the abrasive wear mechanism transferred from the micro-ploughing to the micro-cutting. As shown in Figure 9a–l, the abrasive wear grooves on the worn surface of the nanocomposites became shallower and narrower with the increase in nano-TiC particles content. The penetrating ability of the abrasive Al_2_O_3_ particles increased with the applied load increasing, which contributed to increasing the depth and width of the grooves [43,44]. As the applied load increased from 5 N, to 15 N, to 25 N, the amount of abrasive debris and the plastic deformation regions along the grooves on the worn surface of the Al-Cu alloy and nanocomposites increased as shown in Figure 9a–l. The penetration depth and the micro-cutting efficiency of the abrasive Al_2_O_3_ particle decreased with the decrease in the sizes of abrasive Al_2_O_3_ particle, while the abrasive wear resistance of the samples increased. As observed in Figure 9l,p,t, with the abrasive Al_2_O_3_ particle sizes decreasing from 23 μm, to 13 μm, to 6.5 μm, the width and depth of the grooves decreased obviously and the shallow and narrow grooves occupied the main parts. The micro-cutting was the abrasive wear mechanism for the Al-Cu alloy and the nanocomposites tested under the abrasive Al_2_O_3_ particle sizes of 13 μm and 6.5 μm.

**Figure 10:**
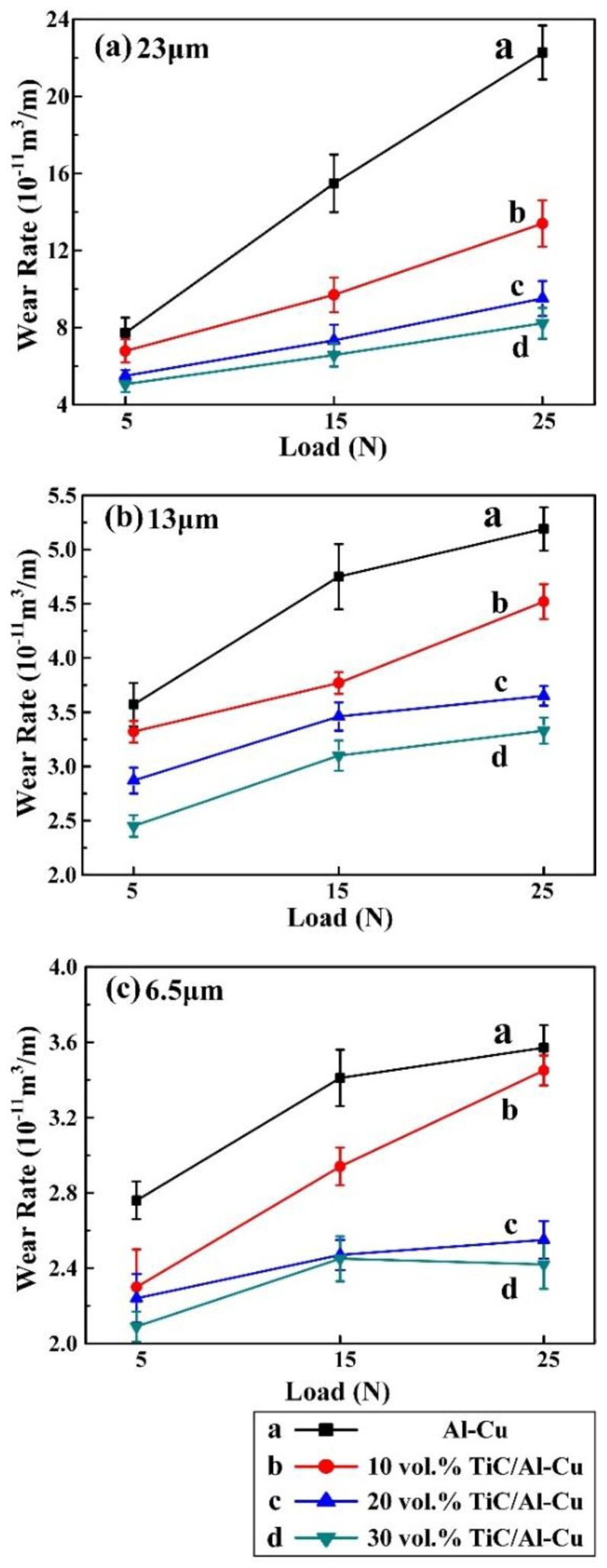
Variations of wear rate with applied load for the Al-Cu alloy and TiC/Al-Cu nanocomposites (fabricated by the pure C black) under the abrasive Al_2_O_3_ particle sizes of (**a**) 23 μm, (**b**) 13 μm, and (**c**) 6.5 μm.

**Figure 11:**
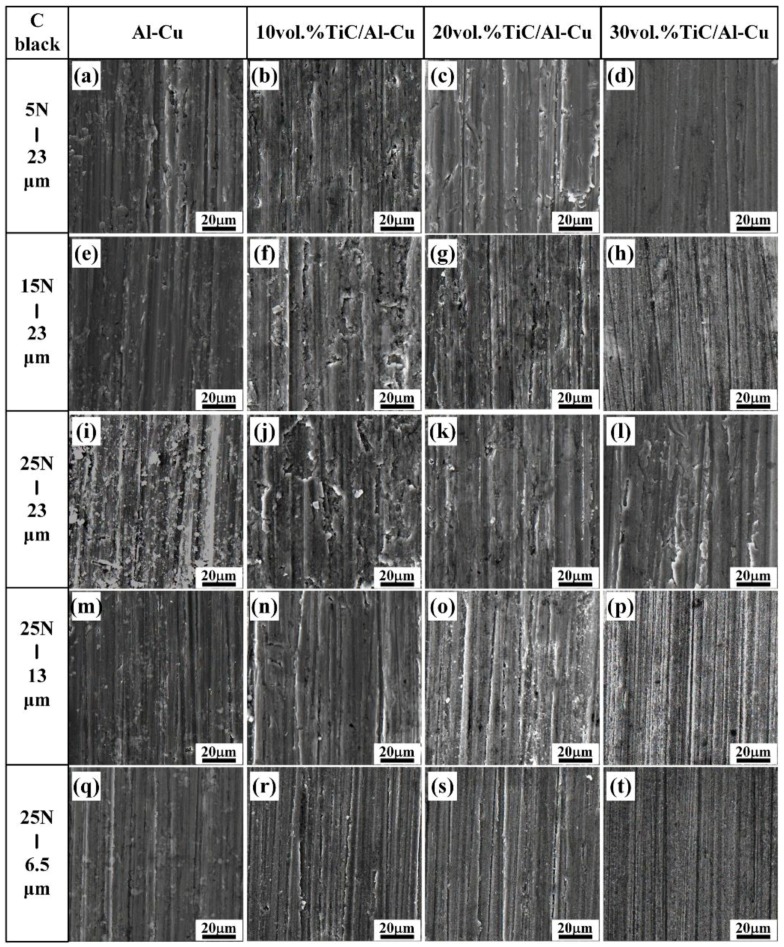
The SEM micrographs of worn surface of the Al-Cu alloy and TiC/Al-Cu nanocomposites (fabricated by the pure C black) under various abrasive Al_2_O_3_ particle sizes and applied loads. (**a**) Al-Cu alloy, (**b**) 10B, (**c**) 20B and (**d**) 30B under tested load of 5 N and abrasive Al_2_O_3_ particle size of 23 μm; (**e**) Al-Cu alloy, (**f**) 10B, (**g**) 20B and (**h**) 30B under tested load of 15 N and abrasive Al_2_O_3_ particle size of 23 μm; (**i**) Al-Cu alloy, (**j**) 10B, (**k**) 20B and (**l**) 30B under tested load of 25 N and abrasive Al_2_O_3_ particle size of 23 μm; (**m**) Al-Cu alloy, (**n**) 10B, (**o**) 20B and (**p**) 30B under tested load of 25 N and abrasive Al_2_O_3_ particle size of 13 μm; (**q**) Al-Cu alloy, (**r**) 10B, (**s**) 20B and (**t**) 30B under tested load of 25 N and abrasive Al_2_O_3_ particle size of 6.5 μm.

Figure 10 shows the variations of wear rate with applied load (5 N, 15 N, and 25 N) for the Al-Cu matrix alloy and in situ TiC/Al-Cu nanocomposites (10B, 20B, and 30B) under various abrasive Al_2_O_3_ particle sizes. The WR of the nanocomposites increased with the increases in the applied load and abrasive Al_2_O_3_ particle size, while decreased with the nano-TiC particles’ content increasing. As the applied load increased from 5 N, to 15 N, to 25 N, the WR of the nanocomposite 30B increased from 5.06, 6.58 to 8.22 (10^−11^ m^3^/m) against the abrasive Al_2_O_3_ particle size of 23 μm. When the applied load increasing up to 25N, the abrasive wear resistance of the nanocomposite 30B was 1.71, 0.56, and 0.48 times higher than those of the Al-Cu alloy with the abrasive Al_2_O_3_ particle size 23 μm, 13 μm, and 6.5 μm, respectively. Figure 11 shows the worn surface images of the Al-Cu alloy and the nanocomposites 10B, 20B and 30B. It can be observed in Figure 11 that the worn surface of the nanocomposites 10B, 20B, and 30B displayed similar variation tendencies with the nanocomposites 10H, 20H, and 30H, as a function of the applied load and abrasive particle size. 

**Figure 12:**
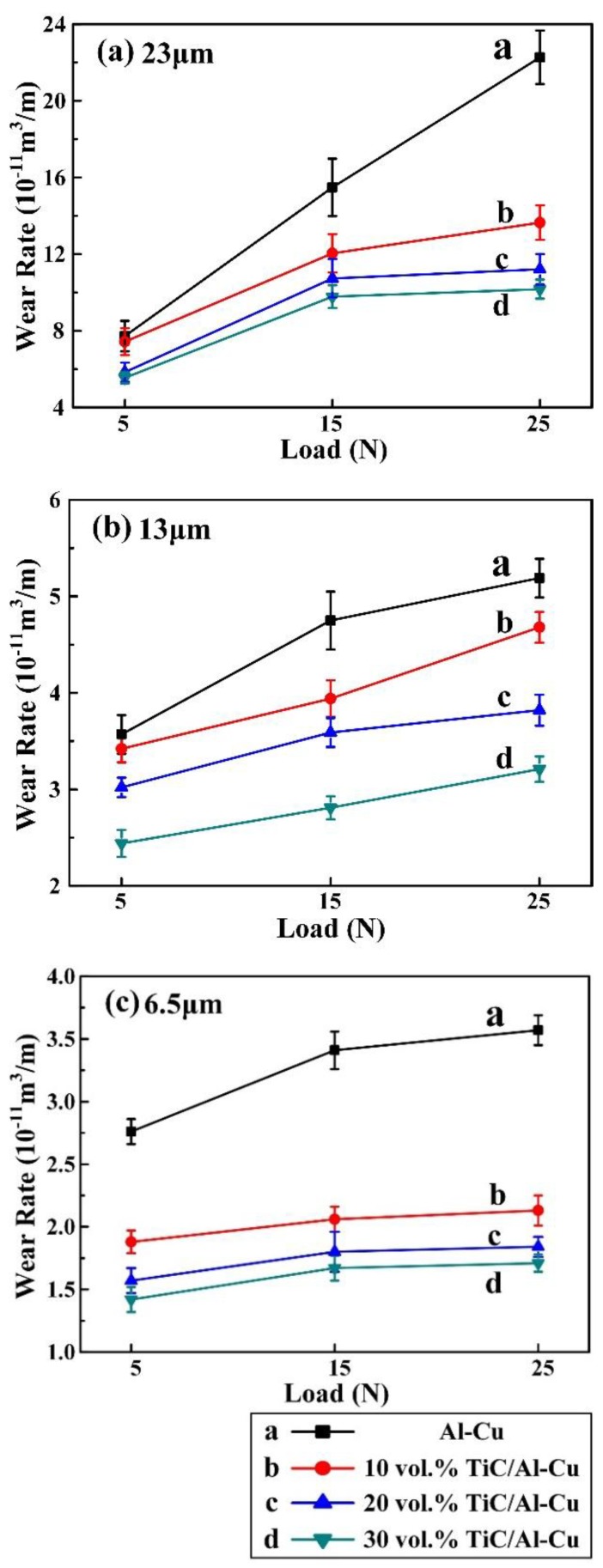
Variations of wear rate with applied load for the Al-Cu alloy and TiC/Al-Cu nanocomposites (fabricated by the pure CNTs) under the abrasive Al_2_O_3_ particle sizes of (**a**) 23 μm, (**b**) 13 μm, and (**c**) 6.5 μm.

**Figure 13:**
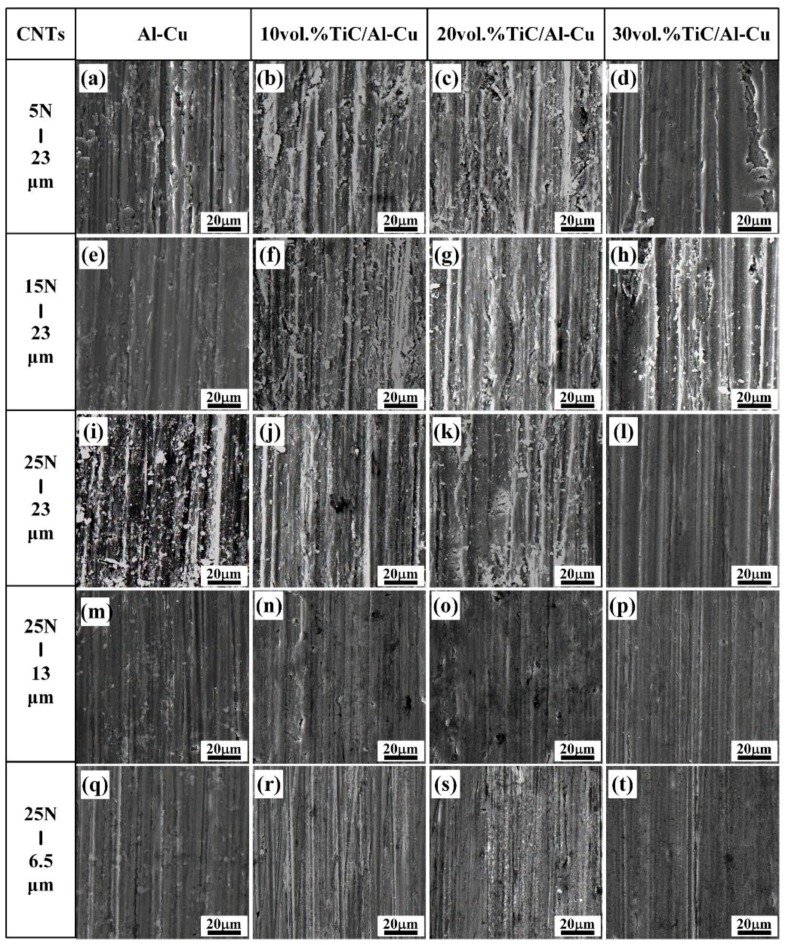
The SEM micrographs of worn surface of the Al-Cu alloy and TiC/Al-Cu nanocomposites (fabricated by the pure CNTs) under various abrasive Al_2_O_3_ particle sizes and applied loads. (**a**) Al-Cu alloy, (**b**) 10C, (**c**) 20C and (**d**) 30C under tested load of 5 N and abrasive Al_2_O_3_ particle size of 23 μm; (**e**) Al-Cu alloy, (**f**) 10C, (**g**) 20C and (**h**) 30C under tested load of 15 N and abrasive Al_2_O_3_ particle size of 23 μm; (**i**) Al-Cu alloy, (**j**) 10C, (**k**) 20C and (**l**) 30C under tested load of 25 N and abrasive Al_2_O_3_ particle size of 23 μm; (**m**) Al-Cu alloy, (**n**) 10C, (**o**) 20C and (**p**) 30C under tested load of 25 N and abrasive Al_2_O_3_ particle size of 13 μm; (**q**) Al-Cu alloy, (**r**) 10C, (**s**) 20C and (**t**) 30C under tested load of 25 N and abrasive Al_2_O_3_ particle size of 6.5 μm.

Figure 12 shows the variations of wear rate with applied load (5 N, 15 N, and 25 N) for the Al-Cu matrix alloy and in situ TiC/Al-Cu nanocomposites (nanocomposites 10C, 20C, and 30C) under various abrasive Al_2_O_3_ particle sizes. Figure 13 shows the worn surface of the Al-Cu alloy and nanocomposites 10C, 20C, and 30C against various applied loads and abrasive Al_2_O_3_ particle sizes. As seen in Figure 12 and Figure 13, the nanocomposite 30C showed lower WR and smoother worn surface at the tested conditions. With the applied load increasing from 5 N, 15 N to 25 N, the WR of the nanocomposite 30C increased from 5.55, 9.78 to 10.17 (10^−11^ m^3^/m) against the abrasive Al_2_O_3_ particle size of 23 μm. When the applied load was 25 N, the abrasive wear resistance of the nanocomposite 30C was 1.19, 0.85 and 1.08 times higher than that of the Al-Cu base alloy with the abrasive Al_2_O_3_ particle size 23 μm, 13 μm, and 6.5 μm, respectively. It can be inferred that abrasive wear of the nanocomposites 10C, 20C, and 30C showed a similar variation tendency with the nanocomposites 10H, 20H, and 30H and nanocomposites 10B, 20B, and 30B under the effects of different applied load and abrasive Al_2_O_3_ particle size.

**Figure 14:**
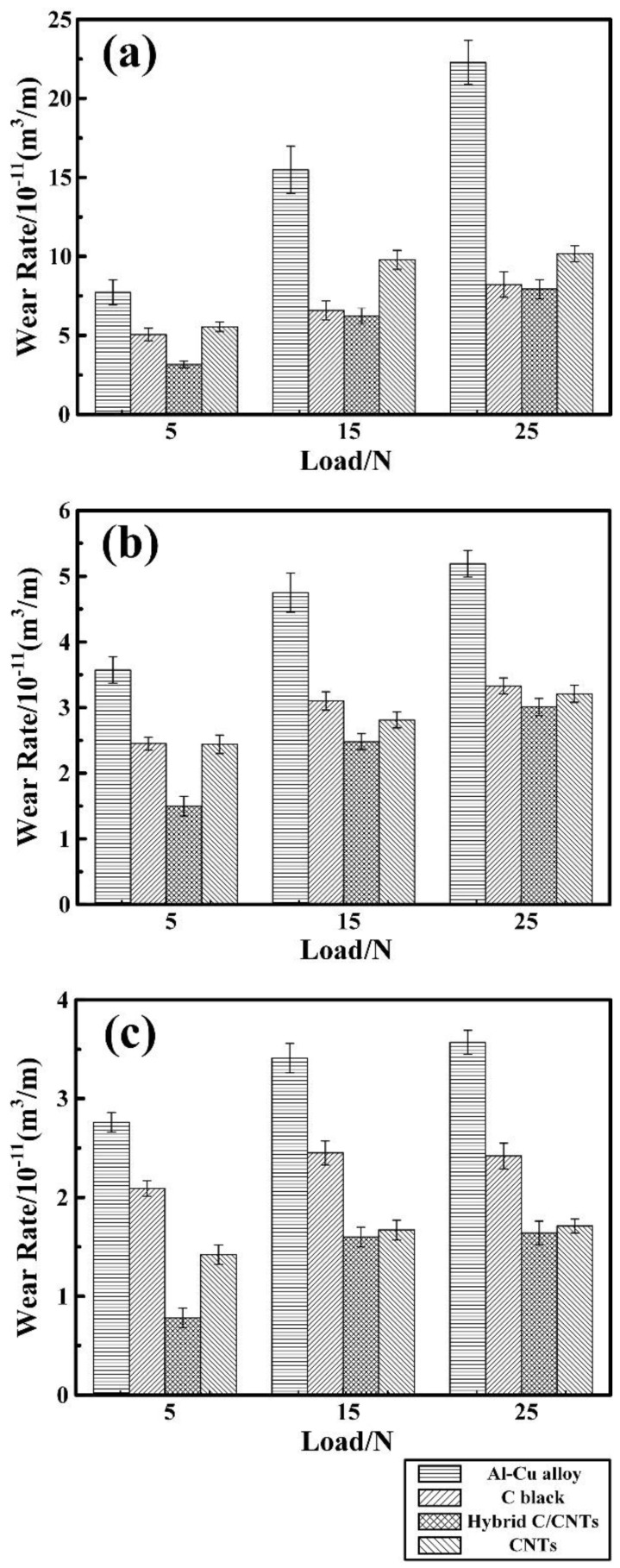
The comparisons in wear rate vs. applied load of the 30 vol.% TiC/Al-Cu nanocomposites fabricated by different carbon sources tested under abrasive Al_2_O_3_ particle size of (**a**) 23 μm, (**b**) 13 μm, and (**c**) 6.5 μm.

**Figure 15:**
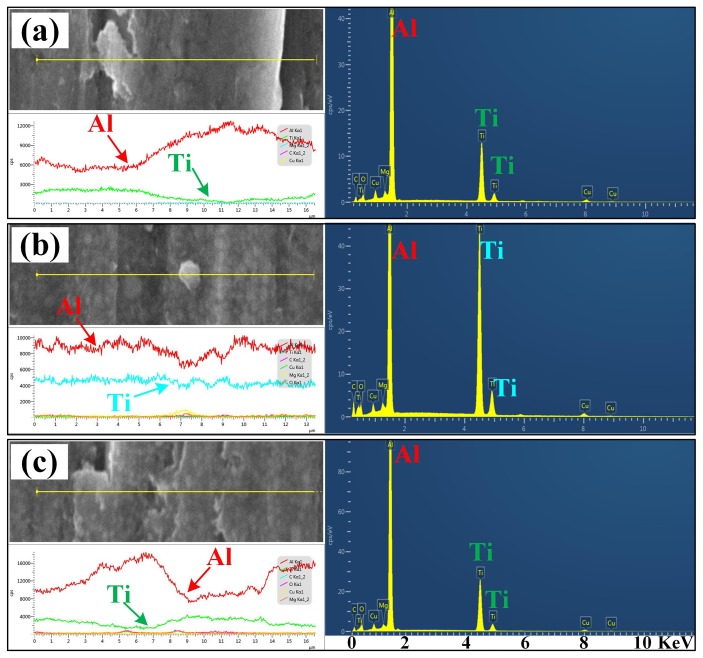
SEM images of worn surface and corresponded EDS analysis results of 30 vol.% TiC/Al-Cu nanocomposites fabricated by carbon source of (**a**) C black, (**b**) hybrid C/CNTs, and (**c**) CNTs.

Figure 14 shows the comparisons in wear rate vs. applied load of the nanocomposites 30B, 30H, and 30C against the abrasive Al_2_O_3_ particle size of 23 μm, 13 μm, and 6.5 μm. It can be observed that the abrasive wear rates of the nanocomposite 30H were lower than those of the nanocomposites 30B and 30C at all the tested conditions. The nanocomposite 30H obtained higher abrasive wear resistance than that of the nanocomposites 30B and 30C. Figure 15 shows the worn surfaces and corresponded EDS analysis results of the nanocomposites 30B, 30H, and 30C, which tested against the abrasive Al_2_O_3_ particle size of 23 μm and applied load of 5N. The distribution of Ti element in the EDS analysis result indicated the dispersion of the nano-TiC particles in the nanocomposites. As observed, the distribution of Ti in nanocomposite 30H was much more homogenous than that in the nanocomposites 30B and 30C. The nanocomposite 30H obtained much more homogenous distribution of nano-TiC particles than that of the nanocomposites 30B and 30C, which was consistent with the results as shown in Figure 3. It can be inferred that the better abrasive wear resistance of the nanocomposites 30H than those of the nanocomposites 30B and 30C was attributed to the much more homogenously-distributed nano-TiC particles in the nanocomposite 30H.

**Figure 16:**
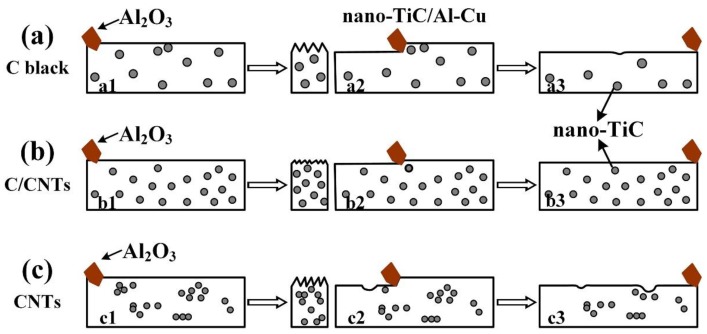
The abrasive wear behavior diagrams of TiC/Al-Cu nanocomposites fabricated by carbon source of (**a**) C black (nanocomposite 30B), (**b**) hybrid C/CNTs (nanocomposite 30H), and (**c**) CNTs (nanocomposite 30C).

Figure 16 shows the abrasive wear behavior diagrams of 30 vol.% TiC/Al-Cu nanocomposites fabricated by different carbon sources. The nano-TiC particles played the role of load-supporting elements and increased the hardness of the TiC/Al-Cu nanocomposites. The penetration depth decreased as the hardness of the nanocomposites increased in the stages of a1, b1, and c1. As the abrasive wear proceeded, the abrasive Al_2_O_3_ particles penetrated into the substrate and converted the materials into the debris and chips (stages a2, b2, and c2). The abrasive wear loss of the nanocomposites occurred during the reciprocating movements of the abrasive Al_2_O_3_ particle (stages a3, b3, and c3). The grooves and the wear debris formed on worn surface, as shown in Figure 9, Figure 11 and Figure 13. As mentioned above, the nano-TiC particles obtained excellent interfacial bonding with the Al matrix, which obviously increased the strength and hardness of nanocomposites. It can be inferred that the differences in the abrasive wear resistance of the nanocomposites 30B, 30H, and 30C were attributed to the sizes and distributions of the nano-TiC particles. The number of nano-TiC particles in the nanocomposite 30B was smaller than that in the nanocomposite 30H due to the bigger average size of nano-TiC particles in the nanocomposite 30B (239 nm) than that in the nanocomposite 30H (176 nm). As the number of nano-TiC particles increased, the direct contacting surface between the abrasive Al_2_O_3_ particles and the Al substrate reduced which cause the decreased abrasive wear rates of the nanocomposites. The nanocomposite 30H showed better abrasive wear resistance than that of the nanocomposite 30B. It is necessary to mention that the dispersions of nano-TiC particles in the nanocomposite 30C was not ideal homogenous. The clusters of nano-TiC particles were expected to act as the preferential sites for crack nucleation, which inclined to form spalling pits and increased the wear rates of the nanocomposite at the stage of c3, as shown in Figure 16c. The local softened Al matrix without enhancers of nano-TiC particles was easily removed by the micro-ploughing and micro-cutting behaviors of the abrasive Al_2_O_3_ particles. The best abrasive wear resistance of nanocomposite 30H was attributed to the high amount of TiC particles and the homogenous distribution of nano-TiC particles.

## 4. Conclusions

The in situ TiC/Al-Cu nanocomposites were synthesized in the Al-Ti-C systems with various carbon sources (pure C black, hybrid C/CNTs, and pure CNTs) by the combined method of combustion synthesis, hot pressing and hot extrusion. The average sizes of nano-TiC particles ranged from 67 nm to 239 nm in the fabricated nanocomposites. The sizes of synthesized nano-TiC particles increased with the TiC particles content increasing, while decreased with the transfer of carbon source from pure C black, hybrid C/CNTs to pure CNTs. The TiC/Al-Cu nanocomposites fabricated by the hybrid C/CNTs obtained more homogenously distributed nano-TiC particles than that in the nanocomposites synthesized by pure C black and pure CNTs. The 30 vol.% TiC/Al-Cu nanocomposite (nanocomposite 30H) fabricated by the hybrid C/CNTs exhibited the best comprehensive tensile properties (yield strength (531 MPa), tensile strength (656 MPa), fracture strain (3.0%), and the highest hardness (331.2 HV). The superior tensile properties and abrasive wear resistance of the nanocomposite 30H were attributed to the higher nano-TiC particles content, better Orowan strengthening effects, much more homogenously-dispersed nano-TiC particles, and excellent Al-TiC interface bonding.

## Figures and Tables

**Table 1:** Characteristics of the in situ TiC/Al-Cu nanocomposites synthesized in Al-Ti-C systems.

Samples	Designed Composition	Actual TiC Content	Carbon Source	Used Powders (wt.%)
10B	10 vol.% TiC/Al-Cu	8.9 vol.%	C black	83.1% Al + 13.5% Ti + 3.4% C black
10H	10 vol.% TiC/Al-Cu	9.0 vol.%	Hybrid C/CNTs	83.1% Al + 13.5% Ti + 1.7% C black + 1.7% CNTs
10C	10 vol.% TiC/Al-Cu	9.2 vol.%	CNTs	83.1% Al + 13.5% Ti + 3.4% CNTs
20B	20 vol.% TiC/Al-Cu	19.8 vol.%	C black	68.7% Al + 25.0% Ti + 6.3% C black
20H	20 vol.% TiC/Al-Cu	19.9 vol.%	Hybrid C/CNTs	68.7% Al + 25.0% Ti + 3.15% C black + 3.15% CNTs
20C	20 vol.% TiC/Al-Cu	19.9 vol.%	CNTs	68.7% Al + 25.0% Ti + 6.3% CNTs
30B	30 vol.% TiC/Al-Cu	29.8 vol.%	C black	56.1% Al + 35.1% Ti + 8.8% C black
30H	30 vol.% TiC/Al-Cu	29.9 vol.%	Hybrid C/CNTs	56.1% Al + 35.1% Ti + 4.4% C black + 4.4% CNTs
30C	30 vol.% TiC/Al-Cu	29.9 vol.%	CNTs	56.1% Al + 35.1% Ti + 8.8% CNTs

**Table 2:** Room tensile properties and hardness of the Al-Cu matrix alloy and the TiC/Al-Cu nanocomposites.

Samples	*σ*_0.2_ (MPa)	*σ*_UTS_ (MPa)	*ε*_f_ (%)	Hardness (HV)	Actual Density (g/cm^−3^)
Al alloy	320 ± 6	468 ± 13	17.6 ± 2.8	135.7 ± 3	2.997 ± 0.001
10B	394 ± 8	493 ± 12	2.5 ± 1.4	213.8 ± 5	2.814 ± 0.003
10H	441 ± 6	570 ± 16	2.4 ± 0.8	228.5 ± 8	3.249 ± 0.002
10C	415 ± 7	553 ± 12	3.9 ± 2.0	199.3 ± 5	3.085 ± 0.002
20B	426 ± 5	522 ± 10	2.3 ± 0.5	270.0 ± 4	3.293 ± 0.004
20H	503 ± 8	636 ± 13	3.2 ± 0.6	295.6 ± 9	3.575 ± 0.002
20C	471 ± 9	591 ± 8	3.0 ± 1.3	269.9 ± 11	3.320 ± 0.003
30B	470 ± 10	551 ± 9	2.3 ± 0.2	303.3 ± 5	3.538 ± 0.004
30H	531 ± 9	656 ± 12	3.0 ± 0.8	331.2 ± 5	3.555 ± 0.003
30C	508 ± 7	616 ± 14	2.5 ± 0.8	285.1 ± 6	3.599 ± 0.003
